# Enabling In Vivo Longitudinal Evaluation of Descemet's Membrane Thickness in Wild-type and FECD Mice Using Self-Referenced Optical Coherence Microscopy

**DOI:** 10.1167/iovs.66.12.60

**Published:** 2025-09-25

**Authors:** Hadiya Farhath Pattan, Subashree Murugan, Rajalekshmy Shyam, Patrice Tankam

**Affiliations:** 1 School of Optometry, Indiana University, Bloomington, Indiana, United States; 2 VisionFirst Indiana Lions Eye Bank, Carmel, Indiana, United States; 3 Department of Anatomy and Cell Biology, University of Iowa, Iowa City, Iowa, United States

**Keywords:** Fuchs endothelial corneal dystrophy (FECD), double mutant FECD mouse model, Descemet's membrane thickness, optical coherence microscopy (OCM), self-referenced interference

## Abstract

**Purpose:**

Descemet's membrane (DM) remodeling is a key factor in the etiology of early-onset Fuchs endothelial corneal dystrophy (FECD). Although various mouse models have been developed to replicate major FECD phenotypes, including DM thickening, there is currently no imaging technique capable of evaluating changes in mice DM thickness in vivo. This work proposed a novel self-referenced optical coherence microscope (OCM) to longitudinally evaluate age-dependent and FECD-dependent changes in DM thickness in mice.

**Methods:**

The self-referenced OCM used the mouse corneal surface as the reference to mitigate artifacts from breathing-induced motion, steep corneal curvature, and dispersion mismatch, that often compromise the delineation of the DM in vivo. The approach was validated by longitudinally evaluating the DM thickness in four wild-type (WT) and five FECD mice at two time points—5 weeks of age and 16 weeks of age.

**Results:**

Unlike standard OCM, the self-referenced approach enabled the delineation of the DM with an axial resolution of 1.6 µm in the living eyes of WT and FECD mice. A significant increase in DM thickness was observed in FECD mice (2.74 ± 0.12 µm) compared with WT mice (1.85 ± 0.22 µm) at 5 weeks of age. A similar trend was observed at 16 weeks of age (3.20 ± 0.20 µm in FECD mice vs 2.21 ± 0.32 µm in WT mice). No age-dependent increase in DM thickness was observed in either group between 5 weeks of age and 16 weeks of age.

**Conclusions:**

This study represents the first longitudinal in vivo evaluation of age-dependent changes in DM thickness in both WT and FECD mice using self-referenced OCM.

The corneal endothelium consists of a monolayer of hexagonal cells that facilitate both the influx of nutrient-enriched fluid from the aqueous humor into the stroma and the efflux of excess fluid from the stroma, thereby ensuring a constant state of dehydration necessary for maintaining the corneal transparency.^1^^–^^4^ Loss of corneal endothelial cells (CECs) can comprise this hydration regulation, leading to corneal opacity.^2^^,^^4^^–^^6^ Although 0.6% of CECs are lost every year owing to aging without a significant impact on endothelial function,^1^^,^^7^ a significant decrease in CEC density owing to disease conditions can severely impair the pump function of the endothelium, leading to vision loss.^1^^,^^5^ Fuchs endothelial corneal dystrophy (FECD) is one of the primary causes of endothelial dysfunction and a leading indication for corneal transplantation in the United States and many parts of the world.^8^^–^^10^ Current research suggests that FECD is associated with progressive endothelial cell apoptosis, variations in cell size and shape, decreased cell density, increase in Descemet's membrane (DM) thickness, guttae formation associated with extracellular matrix deposits, and stromal edema.^8^^,^^11^^–^^14^ FECD is a multifactorial disorder, with symptoms manifesting at different stages of the disease, making its pathogenesis challenging to understand.^8^^,^^15^

Several genes, including Solute Carrier family 4, sodium bicarbonate transporter member 11 (*SLC4A11*), and alpha 2 collagen VIII (*COL8A2*), have been reported to be implicated in FECD pathogenesis.^12^^,^^16^^,^^17^ Although *COL8A2* knockin mutations are associated with early-onset FECD,^11^ decreased SLC4A11 levels are reported in FECD patients regardless of the associated mutations.^14^^,^^18^^,^^19^ Significant efforts have been made to replicate FECD human phenotypes in genetic mouse models to better understand its pathogenesis.^8^^,^^15^

Jun et al.^11^ have developed a *Col8a2* knock-in (*Col8a2^ki/ki^*) mouse model carrying a point mutation causing a glutamine to lysine substitution at amino acid 455 (*Q455K*) in the alpha 2 collagen VIII gene, which is homologous to the human variant. The model replicated several early-onset FECD phenotypes, including decreased CEC density, guttae formation, and DM thickening and softening. Particularly, their histology study reported an approximately linear age-dependent increase in DM thickness in *Col8a2^ki/ki^* mice ranging from 1.12 ± 0.22 µm at 2 weeks to 4.19 ± 1.17 µm at 24 months of age. The DM thickness at 6 weeks and 16 weeks was 1.83 ± 0.34 µm and 2.19 ± 0.40 µm, respectively. This model, however, did not exhibit stromal edema, a key characteristic of FECD.

In contrast, Ogando et al.^20^ developed a tamoxifen-inducible *Slc4a11* knockdown (*Slc4a11^kd/kd^*) mouse model that replicated key FECD phenotypes such as mitochondrial oxidative stress and increased stromal edema owing to impaired endothelium pump function. However, this model did not exhibit DM thickening or guttae formation.

Our team has recently generated a double mutant FECD mouse model by crossing *Col8a2^ki/ki^* mice with *Slc4a11^kd/kd^* mice to replicate the combined phenotypes of the two mouse models. This double mutant successfully reproduced key features of human FECD, including the increased corneal thickness, decrease in CEC density, and evidence of guttae formation at the later stage.^14^ Additionally, endothelial hexagonality and integrity were compromised, accompanied by increased oxidative stress.^14^

In the study by Jun et al.,^11^ the DM thickness analysis was only possible via transmission electron microscopy on ex vivo end-stage animals owing to limitations with current in vivo imaging systems to resolve the relatively thin DM layer in the eyes of living mice.

Although optical coherence tomography (OCT) has enabled noninvasive in vivo measurement of the thickness of the cornea, epithelium, stroma, and corneal nerves in mice,^21^^–^^24^ its axial resolution is limited for assessing DM thickness, and endothelium cells in vivo.^21^^–^^26^ In addition, artifacts from breathing-induced motion, steep corneal curvature, phase instability, and chromatic dispersion between the reference and sample arms in dual-path OCT systems often contribute to the degradation of the axial resolution in mouse in vivo imaging.^27^^,^^28^ The approach of common-path self-referenced OCT, using a highly reflective layer of the tissue as the reference arm in OCT, was introduced as a strategy for mitigating some of these artifacts.^29^^-^^31^ Our team has recently extended this approach to optical coherence microscopy (OCM) for noncontact in vivo imaging of the human cornea, in which the specular reflection from the corneal surface serves as a reference for the OCM system. This common-path approach, based on self-referenced interference between the highly reflective corneal surface and the underlying corneal structures, effectively mitigated artifacts from dispersion mismatch, corneal curvature, phase instability, and axial motion, thereby improving the axial resolution and image contrast for in vivo imaging of human corneal microstructures.^32^^,^^33^

In the current study, we have extended the approach of self-referenced OCM to in vivo imaging of mice corneas. We longitudinally evaluated age-dependent and FECD-dependent changes in DM and stroma thickness in living wild-type (WT) and double mutant FECD mice.^14^

## Methods

### Generation of Double Mutant Mice

**Figure 1.**
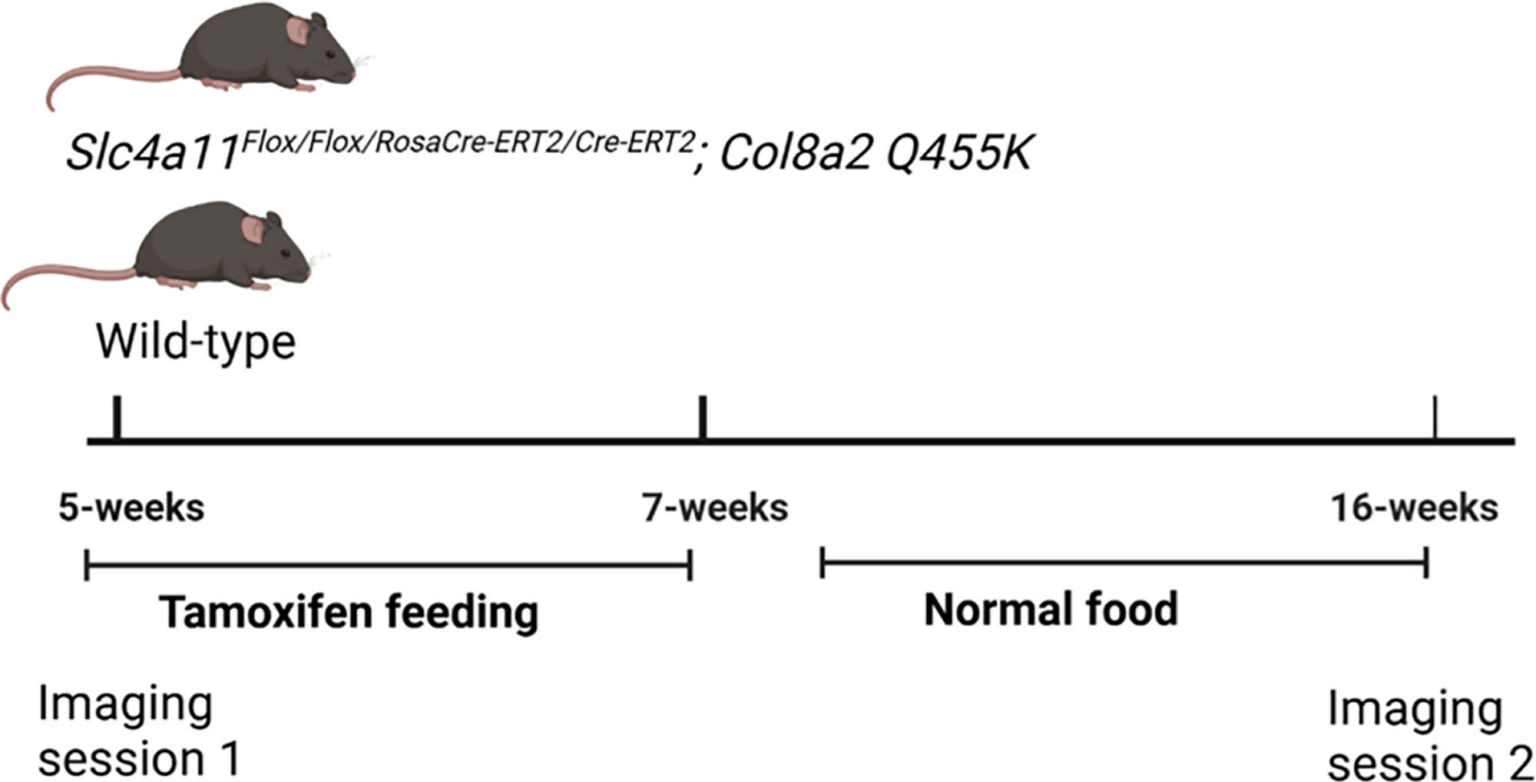
Schematic representation of double mutant FECD mouse model generation and the imaging protocol. WT and FECD mice were fed with tamoxifen from 5 to 7 weeks, after which they were maintained on normal food. Two imaging sessions, one at 5 weeks of age and one at 16 weeks of age, were conducted to evaluate changes to the DM thickness. Image created using BioRender.

All animal procedures were approved by the Institutional Animal Care and Use Committee at Indiana University and adhered to the ARVO statement for the use of animals in Ophthalmic and Vision Science research. The generation of double mutant mice for imaging has been previously described by Murugan et al.^14^ Briefly, double mutant animals (*Col8a2^ki/ki^ (Q455K); Slc4a11^Flox/Flox/RosaCre-ERT2/Cre-ERT2^* or *Col8a2^ki/ki^; Slc4a11^kd/kd^*) were fed with tamoxifen from 5 weeks until 7 weeks of age. We have previously shown that this condition was sufficient to knockdown *Slc4a11* expression (Fig. 1).^14^ Animals were maintained on normal chow until 16 weeks of age (Fig. 1), at which time point we have previously shown that major FECD phenotypes (guttae, corneal edema, and endothelial cell loss) were evident in double mutant animals. Age-matched WT littermates were used as controls. Five FECD and four WT mice were evaluated in this longitudinal study. Two imaging sessions were carried out on animals at 5 weeks of age and 16 weeks of age.

### Custom-Designed In Vivo Imaging System for Mice Cornea

**Figure 2.**
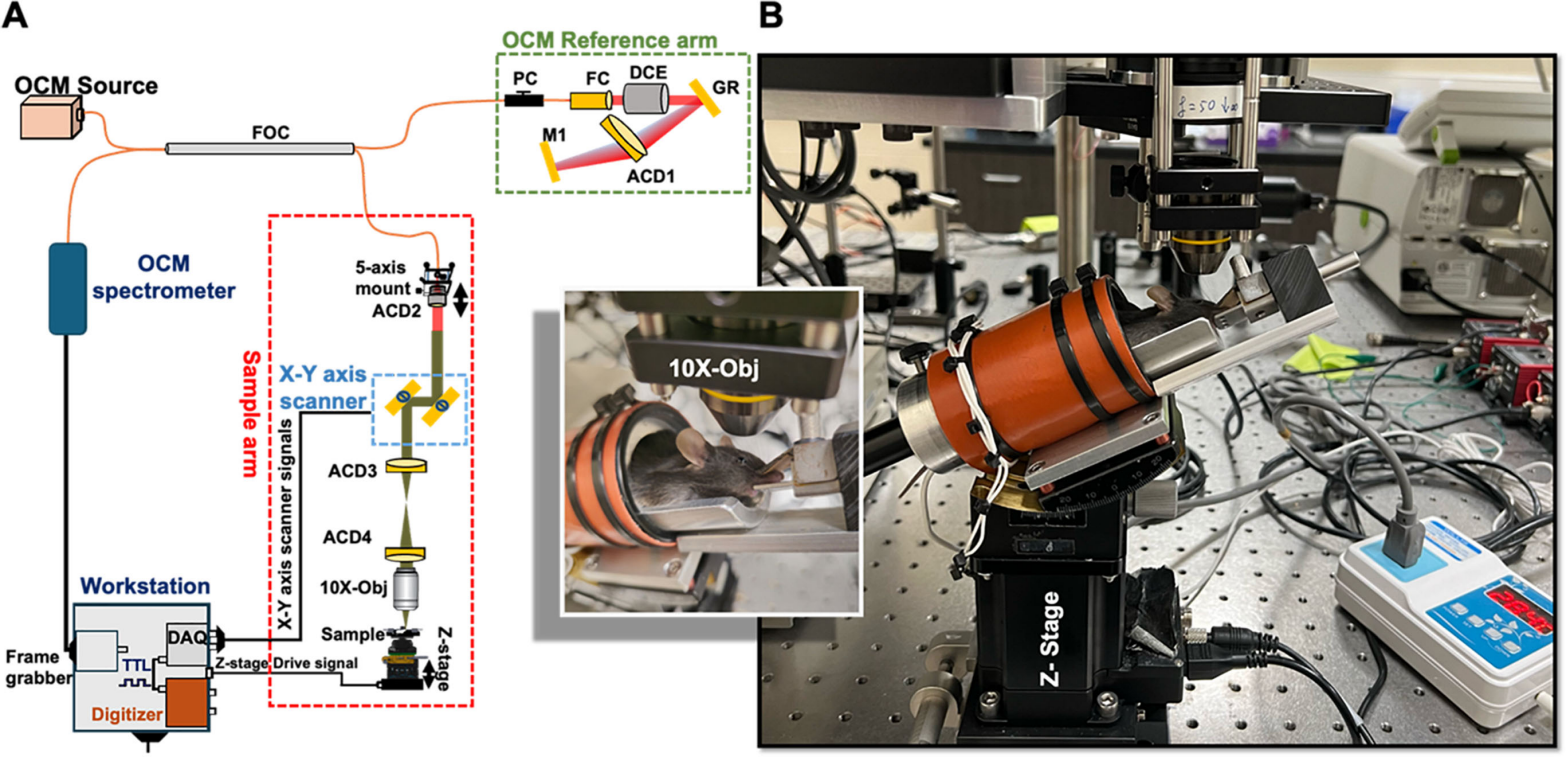
OCM and imaging setup. **(A)** Schematic of the OCM system. ACD, achromatic doublet; DCE, dispersion compensation element; DAQ, data acquisition card; DM, dichroic mirror; FC, fiber collimator; FOC, fiber optics coupler; GR, grating; M, mirror; obj, microscopic objective; PC, polarization controller; TTL, trigger signal. **(B)** Custom-designed mouse holder mounted on a five-axis stage to properly align the mouse's eye to the system and included a heating pad with temperature control. A close-up view of the mouse after positioning and ready to be imaged.

Figure 2A presents the schematic of the custom-designed OCM system. The system was powered with a near-infrared light source with a central wavelength of 850 nm and a spectral bandwidth of 165 nm (M-T-850-HP-I, Superlum, Cork, Ireland), providing a theoretical axial resolution of 1.9 µm in air. The light source was transmitted to the interferometer through a 50/50 fiber optic coupler FOC (TW850R5A2, Thorlabs, Newton, NJ, USA). The reference arm consisted of a polarization controller PC (FPC030, Thorlabs), fiber collimator FC (47-222, Edmund Optics, Barrington, NJ, USA), dispersion compensation element DCE (LSM54DC1, Thorlabs), grating GR (GR25-0608, Thorlabs), achromatic doublet ACD1 (AC254-030-B, Thorlabs), and flat mirror M1 (PFSQ10-03-P01, Thorlabs). The sample arm beam was collimated using a 19-mm achromatic doublet (AC127-019-B-ML, Thorlabs) to match the 6-mm diameter clear aperture of the galvanometric scanning mirrors. The beam transmitted through a dual-axis galvanometric scanner (6215HSM40, Cambridge Technology, Bedford, MA, USA), was expanded (×1.7) via a telescopic system composed of a 30-mm VIS-NIR achromatic doublet (ACD2, #49-352, Edmund Optics) and a 50-mm VIS-NIR achromatic doublet (ACD3, #49-356, Edmund Optics). The expanded beam filled the 10-mm full aperture of a 10× microscopic objective (Olympus UPLFLN 10 × 0.25 NA, Edmund Optics) with a working distance of 10 mm. The interference signal was detected using a customized spectrometer (Cobra-S 800, Wasatch Photonics, Morrisville, NC, USA) equipped with a high-speed line-scan camera operating at 250k A-scans/second (Octoplus 2k, Teledyne e2v, Chelmsford, UK). The data were recorded via a frame grabber (AXN-PC2-CL-1xE, Bitflow, MA, USA).

To mitigate motion artifacts induced by breathing occurring every 1 to 2 seconds under standard anesthetic conditions,^32^^,^^34^^,^^35^ the sample size was reduced from 1000 × 1000 to 500 × 500 A-scans, thus decreasing the volume acquisition time from 4 seconds to 1 second while enabling corneal imaging of a field of view (FOV) of 500 × 500 µm^2^.

The interference between the back-scattered light from the underlying corneal layers and the reflection from the reference arm of OCM (referred to as standard interference), as well as the specular reflection from the corneal surface (referred to as self-referenced interference) that, in contrast, generated a duplicated three-dimensional (3D) image of the cornea. The reference arm was delayed to adjust the spectral separation between the two 3D images from the standard and self-referenced processes. The self-referenced approach was used to evaluate the thickness. For this analysis, the role of the reference arm was minimal, although it was beneficial during the initial alignment of the eye and in visualizing the focusing of the beam into the cornea.

### Evaluating the Axial and Lateral Resolution of the System

The axial resolution in both standard and self-referenced OCM was evaluated by measuring the full width at half maximum (FWHM) of the axial point spread function. A 150-µm-thick glass slide was used as the sample, because this thickness closely approximates that of the mouse cornea. The self-referenced image was generated between the front surface (used as the reference) and the back surface of the glass slide, and the standard reference image was formed from the interference between the reference arm of OCM and the two surfaces of the glass slide. The lateral resolution of the OCM was evaluated using a positive, nonfluorescent USAF 1951 resolution test chart (R1L1S1P, Thorlabs). The 150-µm-thick glass slide was placed over the test chart to generate the self-referenced image between the test chart and the front surface of the glass slide. The lateral resolution was evaluated on both images of the test chart from the self-referenced and standard OCM.

### Imaging Protocol

Individual WT and FECD mice were imaged at two timepoints—5 weeks of age and 16 weeks of age (Fig. 1)—using OCM. For each imaging session, the mice were first anesthetized using a solution containing 100 mg/kg of ketamine and 10 mg/kg of xylazine, administered intraperitoneally, before positioning on the mouse holder.

A deep plane of anesthesia was verified by checking pedal reflexes and ensuring that the anesthetized mice were able to breathe normally throughout the imaging process. The anesthesia lasted for approximately 45 minutes, providing ample time to image both eyes of each mouse. A saline solution was applied to keep the ocular surface moist and smooth, thereby optimizing the specular reflection from the corneal surface, which was necessary to generate optimal self-referenced images. The holder was mounted on a five-axis stage (including three translations and two rotations) allowing to align the cornea of the mouse perpendicular to the microscope objective. The holder included a back block with a gap for the tail to be inserted, preventing the mouse from sliding, while the mouth was secured on a bite bar. The holder also included a heating pad with a temperature controller to maintain the body temperature of the mouse between 27°C and 35°C. Figure 2B presents an example of mouse positioning and alignment with the imaging system.

During the alignment process, the system was first focused on the iris while using real-time cross-sectional views of the eye along the x and y axes, sequentially, with a 2-mm large FOV to center the eye on the system. The system was then focused on the posterior cornea with a reduced FOV of 0.5 mm. Fine alignments were manually performed using the five-axis stage to ensure the corneal surface was relatively perpendicular to the optical axis of the microscope at the center of the FOV. These refinements were essential for enhancing the self-referenced interference signal. Given the limited depth of field of the microscopic objective (∼60 µm), a real-time depth profile at the center of the FOV was used to optimize the focusing of the beam into the DM layer while manually adjusting the Z-stage until a distinct double peak—corresponding with the stroma–DM and DM–endothelium transitions—was visible on the depth profile. For each eye, at least three 3D scans were performed with each 3D scan completed within 1 second. After the imaging session, mice were moved to a water-supplied heating pad and allowed to recover and walk in an isolated warm cage before returning to their housing facility.

### Data Processing and DM Segmentation

OCM volumetric images were processed using custom programs developed in LabVIEW 2017 (National Instruments, Austin, TX, USA) and MATLAB 2023b (The MathWorks, Natick, MA, USA). A MATLAB program was also developed to perform the automated segmentation of the DM on the self-referenced images and compute its thickness map across 3D corneal volumes.

The DM segmentation process involved multiple steps performed in parallel using the *parfor* function in MATLAB. For each 3D data consisting of 500 cross-sectional images of the cornea, a local averaging was first performed on every subset of five consecutive cross-sectional images to reduce the speckle noise, followed by the function *imadjust* in MATLAB to improve the contrast. A 3 × 3 median filter (medfilt2) was then applied to the resulting 496 averaged images to further reduce the noise.

**Figure 3.**
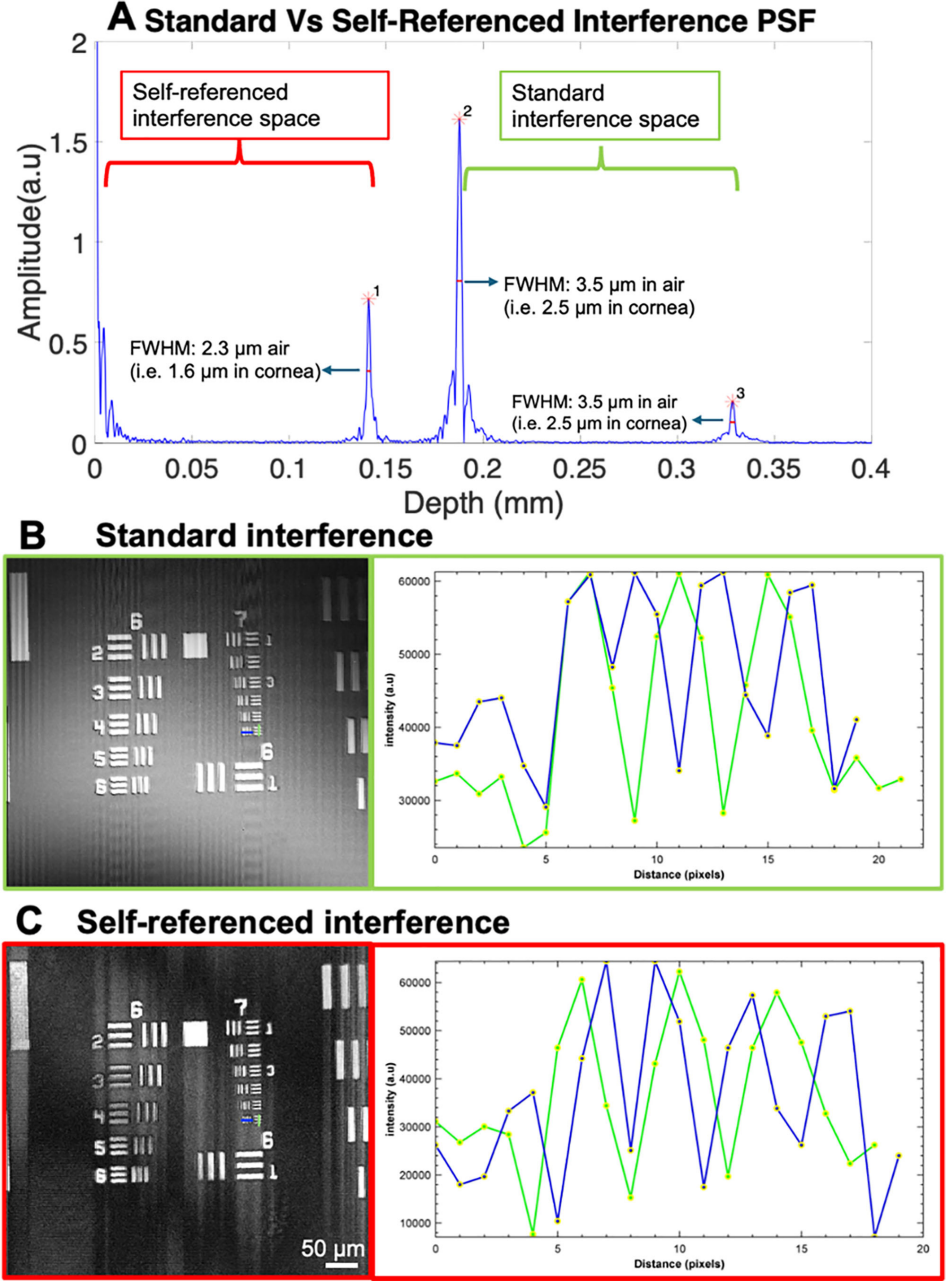
Evaluating the resolution of OCM using the standard and self-referenced interference approaches. **(A)** Depth profile of the 150-µm glass slide, exhibiting three distinct peaks: peak 3 and peak 2 correspond with the front and back surfaces of the glass slide from the standard OCM and peak 1 corresponds with to the back surface of the glass slide from the self-referenced OCM. The measured FWHM for each peak is indicated for air and cornea. **(B**, **C)** Comparable en face images of the USAF 1951 resolution chart over a FOV of 0.5 mm × 0.5 mm from the standard and self-referenced OCM. Intensity profiles along the horizontal (*blue traces*) and vertical (*green traces*) lines demonstrate that both imaging processes can resolve the sixth element of the seventh group of the test chart, corresponding to a lateral resolution of approximately 2 µm.

The *findpeaks* function in MATLAB was used to first detect the peak corresponding with the endothelium and then the second peak corresponding with the transition between the stroma and the DM as illustrated in Figure 3. Peaks were selected based on specific criteria. The first peak was required to lie within the range of 10 microns from the top of the appropriately cropped self-referenced image. The second peak, if present, was constrained within 7 microns from the first peak, with the consideration that the mouse DM is thinner than 7 microns.^11^ Peaks detected beyond these thresholds were excluded.

The difference between the two peaks in terms of pixel number was used to compute the thickness of the DM in microns using a calibration factor of 0.349553 µm/pixel, which accounted for the corneal refractive index (1.387), the number of points (8192 zero-padding) used to compute the Fourier transformation, the spectral resolution of the system defined by the spectral bandwidth of the source (165 nm), and the number of pixels of the line camera (2048). The DM thickness maps were generated for the corresponding FOV of the cornea, with invalid or outlier values represented as NaNs.

The mean and SD values were evaluated for individual mice and different time points. The same process was used for evaluating the stroma thickness by segmenting the third peak corresponding to the Bowman's membrane and measuring its distance relative to the second peak.

### Statistical Analyses

One-way and two-way ANOVAs using GraphPad Prism Software (version 10.0.2; Boston, MA, USA) were performed to evaluate age- and group-dependent differences. A *P* value of 0.05 or less was considered statistically significant.

## Results

### Axial and Lateral Resolution of Self-referenced OCM

Figure 3A presents the depth profile of the glass slide, exhibiting three distinct peaks from the zero order. Peaks 3 and 2 represent the front and back surfaces of the glass, respectively, from the standard OCM. Peak 1 represents the corresponding back surface of the glass slide from the self-referenced OCM.

The FWHM of the three peaks was evaluated to quantify the axial resolution of the system from the standard and self-referenced approaches. The FWHM of peak 3 and peak 2 were evaluated to be 3.5 µm in air, corresponding with 2.5 µm in the cornea. The FWHM of peak 1 was 2.3 µm in air, corresponding with 1.6 µm in the cornea, a factor of 1.5 times better than that of the standard OCM. It is worth noting that, unlike the standard OCM, the self-referenced OCM possesses sufficient axial resolution to resolve a DM thickness of less than 2 µm.

Figures 3B and 3C present comparable en face images of the resolution test chart obtained from standard and self-referenced OCM, respectively, over a FOV of 0.5 mm × 0.5 mm. The intensity profiles along the vertical (green trace) and horizontal (blue trace) axes of the sixth elements of the seventh group of the test chart demonstrate that both interference methods can resolve the sixth element of the seventh group, corresponding to a lateral resolution of approximately 2 µm. Note that the blue traces show four peaks instead of three owing to the instability of the scanner along the *y* axis (i.e., slow axis).

### Comparison of Standard and Self-referenced OCM for DM Thickness Measurements

**Figure 4.**
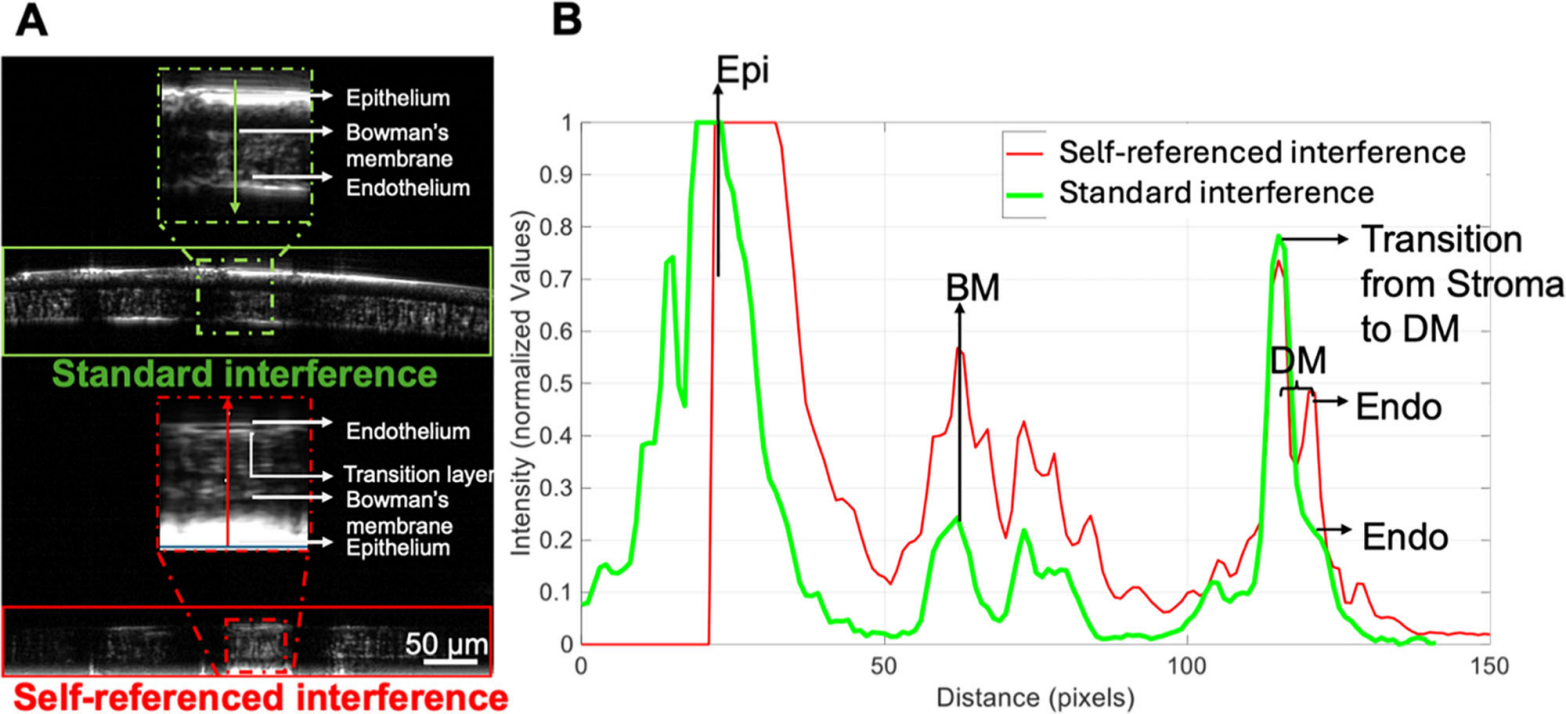
Comparison between standard and self-referenced signals recorded with OCM. **(A)** Cross-sectional images from the standard (*green box*) and self-referenced interference (*red box*) processes. The insets highlight all the layers of the cornea. **(B)** Intensity profile of the same A-scan along the green and red arrows in the insets in **(A)**. Unlike the standard reference image, the self-referenced image can distinctly delineate all peaks of the corneal layers including the stroma/DM transition and the endothelium.

Figure 4A presents simultaneous cross-sectional images of a mouse cornea from the standard (top) and self-referenced (bottom) OCM. Note that the self-referenced image appears to be inverted. The standard interference image was susceptible to artifacts from mouse breathing, steep corneal curvature, and dispersion mismatch, and therefore failed to delineate the DM layer. In contrast, the self-referenced interference image remains highly stable by intrinsically compensating for axial motion. Additionally, this technique inherently compensates for the steep corneal curvature, resulting in a flattened image of the cornea. Finally, the approach is less sensitive to dispersion mismatch inherent in dual-path standard OCM, thus resulting in enhanced axial resolution. This approach significantly enhanced the visibility of the DM layer and other corneal structures.

Note that the saturation observed on the epithelium (Fig. 4A, bottom) arises from a combination of factors, including the zero-order (DC) signal and reflections from the tear film and the saline solution. Figure 4B presents the intensity profiles of the same A-scan obtained from the standard (green trace) and self-referenced (red trace) images, representing the different layers of the cornea. Both profiles present distinct peaks corresponding to the epithelium, Bowman's membrane, and the transition layer from stroma to DM. However, although the endothelium layer was well-distinguished from the DM layer on the self-referenced profile (red trace), it was not well-distinguished on the standard reference profile (green trace) and only appeared as a slight bump. Thus, the self-referenced approach was used for evaluating the DM thickness.

### Segmentation of DM from Self-referenced Images

**Figure 5.**
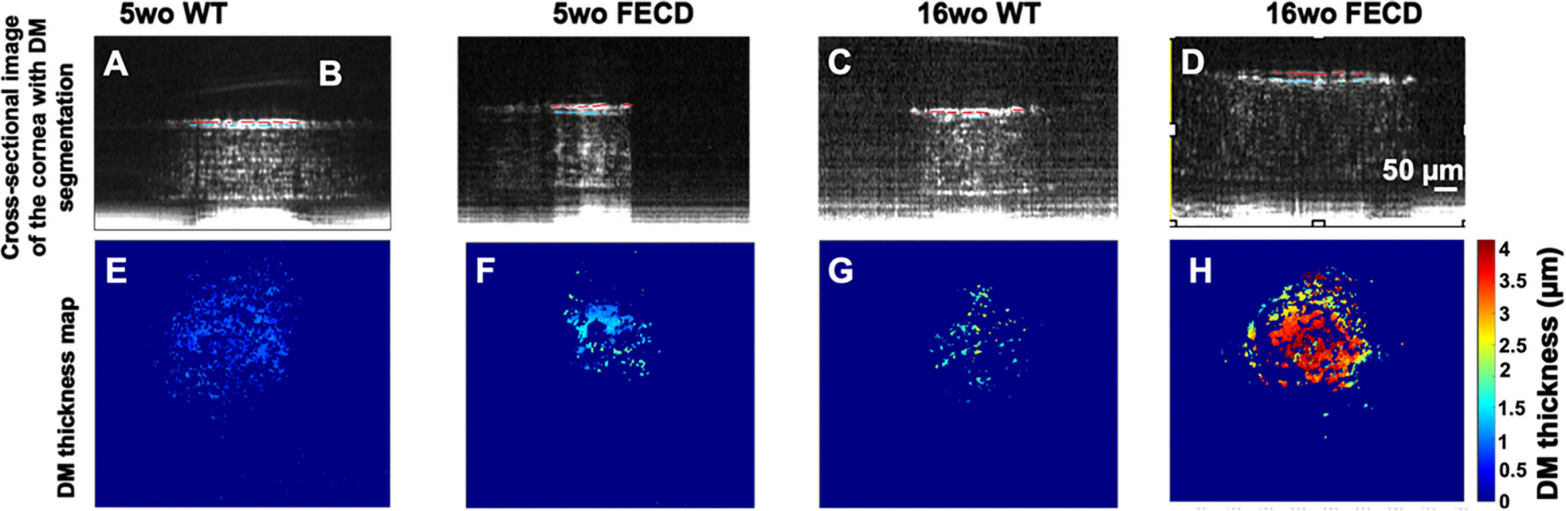
DM thickness evaluation in WT and FECD mice, at different ages. (**A–D**) Cropped self-referenced images of WT and FECD mice at 5 weeks of age and 16 weeks of age. The DM was identified as the gap between the transition signal from the posterior stroma to the DM (*blue line*) and the endothelium (*red line*), with a single representative frame shown out of 500 total frames. (**E**–**H**) Corresponding DM thickness maps across all frames of the cornea. The color bar indicates the range of DM thickness values. The blue background represents outlier values that were excluded.

Figure 5 presents examples of cross-sectional self-referenced images of the cornea (first row) with segmentations of DM stroma transition (red line) and endothelial layer (blue line) in WT and FECD mice at 5 weeks of age and 16 weeks of age. The corresponding DM thickness maps across the FOV of 0.5 mm^2^ (second row) were computed from the 500 frames of the central cornea. Note that the DM thickness appears to be almost uniform in WT compared with FECD mice, in which irregular DM thickening is commonly observed. Notably, FECD mice at 16 weeks of age had increased focal DM thickening compared with other mouse groups.

### Evaluating the Age-dependent Changes in DM and Stroma Thickness in WT and FECD Mice

**Figure 6.**
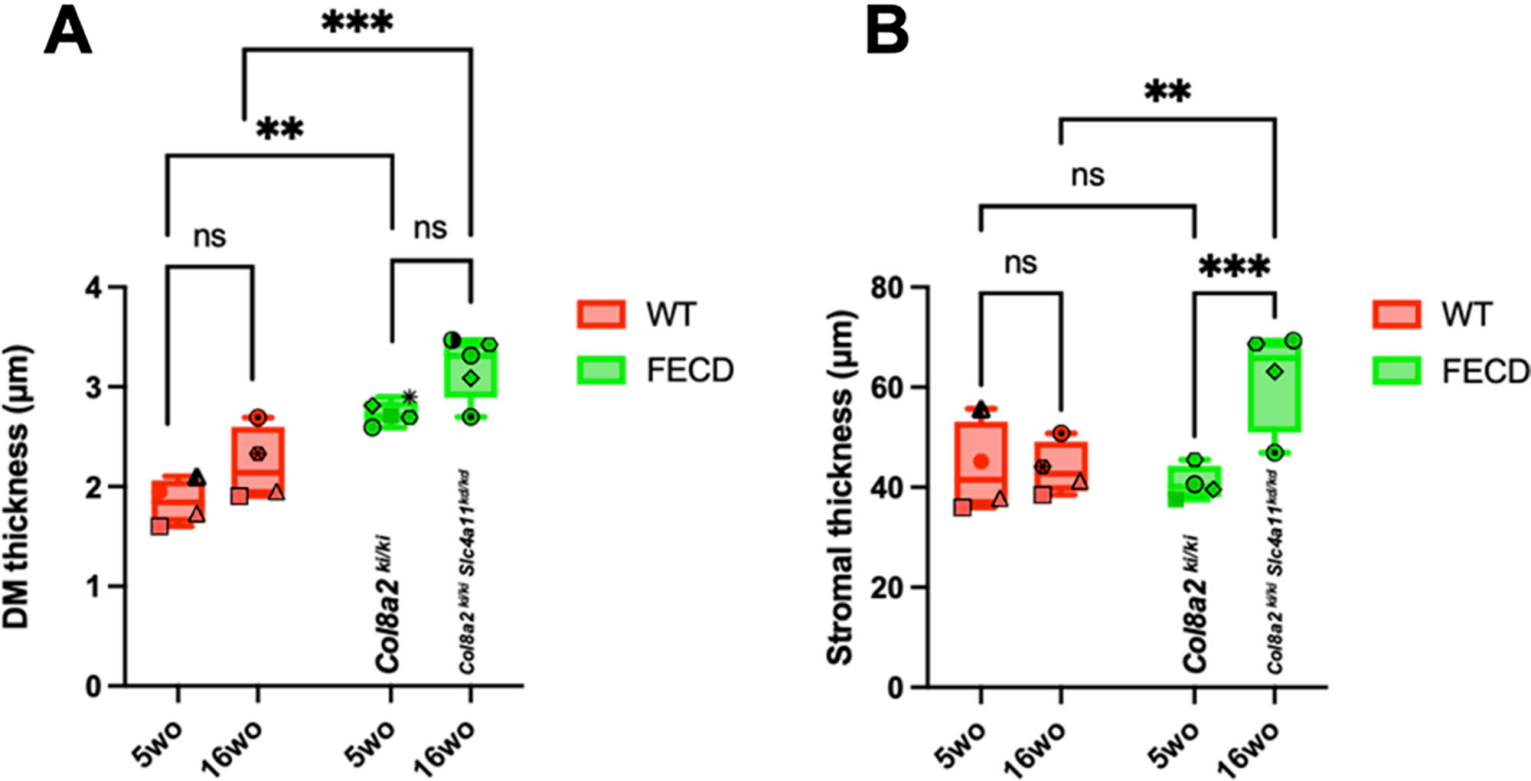
Analysis of DM and stromal thicknesses in WT and FECD mice. **(A)** One-way ANOVA analysis (mean thickness and standard deviation) across four groups 5 weeks of age WT, 5 weeks of age FECD, 16 weeks of age WT, and 16 weeks of age FECD mice. **(B)** Two-way analysis (mean thickness and standard deviation) across four groups 5 weeks of age WT, 5 weeks of age FECD, 16 weeks of age WT, and 16 weeks of age FECD mice. ***P < 0.0002; **P < 0.002; and ns for *P* > 0.05. Matching symbols and colors represent data points from the same animal at different time points, indicating longitudinal inferences.

Figure 6A shows the mean, median, and SD of DM thickness in WT and FECD mice at 5 weeks of age and 16 weeks of age. The DM thickness in Col8a2^ki/ki^ FECD mice (2.74 ± 0.12 µm; *n* = 5) was significantly greater (*P* = 0.0006) than that in WT (1.85 ± 0.22 µm; n = 4) mice at 5 weeks of age. A similar trend (*P* = 0.0001) was observed at 16 weeks of age in double mutant FECD mice (3.20 ± 0.20 µm) compared with WT mice (2.21 ± 0.32 µm). No significant increase in DM thickness was observed between WT mice at 5 weeks of age and at 16 weeks of age (*P* = 0.2023) and between FECD mice at 5 weeks of age and 16 weeks of age (*P* = 0.0599).

Figure 6B presents the mean, median, and SD of the stroma thickness in WT and FECD mice at 5 weeks of age and 16 weeks of age. No statistically significant increase in the stroma thickness was observed between Col8a2^ki/ki^ FECD mice (40.80 ± 3.37 µm) and WT mice (43.66 ± 8.96 µm) at 5 weeks of age (*P* = 0.6021). However, a significant increase (*P* < 0.005) in the stroma thickness was observed in double mutant FECD mice (61.97 ± 10.42 µm) compared with WT mice (43.66 ± 5.25 µm) at 16 weeks of age (*P* = 0.005). These results are consistent with our previous findings of ex vivo characterization of the double mutant FECD mouse model.^14^

## Discussion

Histopathology studies often rely on end-stage ex vivo human samples, restricting insights into disease initiation and progression.^14^ Although animal studies have been broadly adopted for studying developmental and pathological conditions, cross-sectional studies require a large number of animals, increasing breeding and maintenance costs. More important, this approach is subject to interanimal variability that can confound results interpretation. It is also not adequate for tracking progressive changes in the same animal over time. To address these limitations, there is a need for imaging technologies that enable longitudinal assessments of physiological changes in living animals.^36^

Although dual-path OCT systems are commonly used for in vivo evaluation of the thickness of different layers of tissue, the phase instability and dispersion mismatch between the two arms of the interferometer can severely degrade the axial resolution of the system, limiting the capabilities of the system for delineating thin layers of tissues. For instance, our dual-path OCM system using a standard reference mirror achieved an axial resolution of 2.5 µm in the cornea. This resolution was not sufficient to resolve the DM thickness in mice, which was reported to be 1.12 ± 0.22 µm at 2 weeks of age and 1.83 ± 0.34 µm at 6 weeks of age, according to histology studies.^11^

Our group has recently developed a new approach of self-referenced OCM using the corneal surface as the reference mirror to enable in vivo imaging of human corneal microstructures.^32^^,^^33^ Building on this work, the current study extends the utility of the self-referenced approach to longitudinally evaluate age-dependent changes in DM thickness in the living eyes of WT and FECD mice.

The self-referenced approach was used to enhance the axial resolution of the system, by leveraging the highly reflective air–cornea interface of the mouse cornea, thereby enabling interference between the specular reflection from the air–cornea interface and the backscattered light from underlying corneal structures, including the DM and endothelial cells. By eliminating dispersion mismatches inherent in conventional dual-path OCM systems, this method addresses key challenges in imaging thin corneal layers. This approach also enhanced the visibility of thin layers by mitigating motion artifacts typically associated with conventional dual-path OCM systems. Additionally, the self-referenced method further enhanced the visibility of corneal layers by intrinsically flattening the image of the cornea. This approach also holds translational potential for in vivo imaging of human CECs and guttae in healthy and FECD patients.^37^ To accommodate the sensitivity loss of the self-referenced approach owing to the shallow depth of focus of the microscopic objective relative to the human corneal thickness, a custom-designed bifocal lens was integrated into the back aperture of the microscopic objective to generate a dual beam, simultaneously focusing on the corneal surface (reference signal) and the endothelium (sample signal) to enhance the self-referenced signal and enable high-contrast images of endothelial cells and guttae in the living human eye. This study demonstrated that the self-referenced approach could have important prognostic implications in clinical practices, such as monitoring FECD progression and evaluating therapeutic efficacy in clinical trials. The system can be used to evaluate preoperative and postoperative subtle corneal microstructural changes, such as keratocytes degeneration,^38^ or posterior stromal ripples,^39^^,^^40^ which could serve as predictive biomarkers of disease progression or treatment outcomes. For instance, several studies have demonstrated that posterior stromal ripples are associated with an increased risk of graft detachment and could serve as predictive biomarkers of visual recovery after DM endothelial keratoplasty.^39^^,^^40^ The in vivo DM thickness measurements obtained in this pilot study were consistent with existing literature on ex vivo corneas.^11^^,^^41^ In *Col8a2^ki/ki^ Q455K* mice, Jun et al.^11^ reported DM thicknesses of 1.83 ± 0.34 µm at 6 weeks of age and 2.19 ± 0.40 µm at 16 weeks of age. The new FECD mouse model combined *Col8a2^ki/ki^ Q455K* mutation and a tamoxifen-inducible knockdown of *Slc4a11* gene expression. A significant increase in DM thickness was observed in FECD mice compared with WT animals at 5 weeks of age. This change was the result of *Col8a2^ki/ki^* alone; *Slc4a11* knockdown was not effective until week 6, when mice were fed with tamoxifen. At 16 weeks, when *Slc4a11* knockdown was induced and all the major FECD phenotypes were evident, the double mutant FECD mice did not show any significant changes in DM thickness from their baseline levels. This finding indicates that Slc4a11 may not significantly contribute to the increase in DM thickness and the age-dependent changes were not statistically significant between these two timepoints. It is worth noting that these observations should be considered provisional, given the statistically underpowered sample size and the limited sampling of imaging timepoints.

Despite these limiting factors, this study is consistent with the histological findings from Jun et al.,^11^ particularly at 5 weeks of age and 16 weeks of age in WT mice, supporting the validity of the in vivo measurements and demonstrating that COL8A2 may primarily contributes to DM thickening. *Col8a2 Q455K* mice seem to exhibit corneal endothelial dysfunction, but remain otherwise healthy with no developmental abnormalities. In contrast, a study by Ogando et al.^20^ reported that SLC4A11 did not affect DM thickening, but instead primarily impaired pump function. Loss of *SLC4A11* leads to increased oxidative stress, stromal edema, and cell death in the corneal endothelium without any presence of guttae. Consistent with these studies, we observed a 67.5% increase in DM thickness of FECD mice when compared with age-matched WT animals at 5 weeks of age (baseline) before Slc4a11 knockdown. This difference remains unchanged at 16 weeks of age after *Slc4a11* knockdown, suggesting that the loss of *Slc4a11* may not exacerbate the increase in DM thickness. Whether the trend continues past 16 weeks is uncertain. The long-term effect of *Slc4a11* knockdown on DM thickness will be further investigated in future studies. No significant increase in DM thickness was observed from 5 weeks of age to 16 weeks of age in both WT and FECD mouse models. However, a larger sample size, denser sampling timepoints, and longer timespans are necessary to confirm the age-dependent DM changes in WT and FECD mice.

Similarly, stromal edema—a characteristic feature of FECD—is directly linked to reduced lactate efflux from the stroma owing to decreased CEC density. Consistent with findings from our previous studies on corneal thickness changes using pachymetry, we observed no significant increase in stromal edema between double mutant FECD and WT mice at 5 weeks of age. By 16 weeks of age, however, stromal edema was significantly increased in the double mutant FECD mice compared with WT mice, likely owing to compromised pump activity. There was no significant increase in stromal edema from 5 weeks of age to 16 weeks of age in WT animals, suggesting that the stromal thickness remains stable during this developmental period. These results are also consistent with human studies, where stromal edema is observed in the later stages of the disease, suggesting that stromal edema occurs as a secondary effect owing to CEC loss rather than as a primary consequence of FECD itself.^14^^,^^42^ Although the current and previous studies provide a temporal window for pump function disruption in the FECD mouse model, we agree that future studies incorporating the in vivo assessment of physiological parameters such as corneal hydration and endothelial pump activity will enable the correlation of structural and functional characteristics, thereby strengthening the translational relevance and applicability of these findings.

Although Figures 4 and 5 show suboptimal cross-sectional image quality compared with contemporary mouse corneal OCT images, it is worth noting that the latter often rely on multiple 2D cross-sectional images captured at the same transverse location, followed by signal averaging to improve image quality. The approach in this study focused on acquiring fast 3D images to generate en face images of different layers. Thus, such averaging process to improve cross-sectional image quality was not performed in this study.

**Figure 7.**
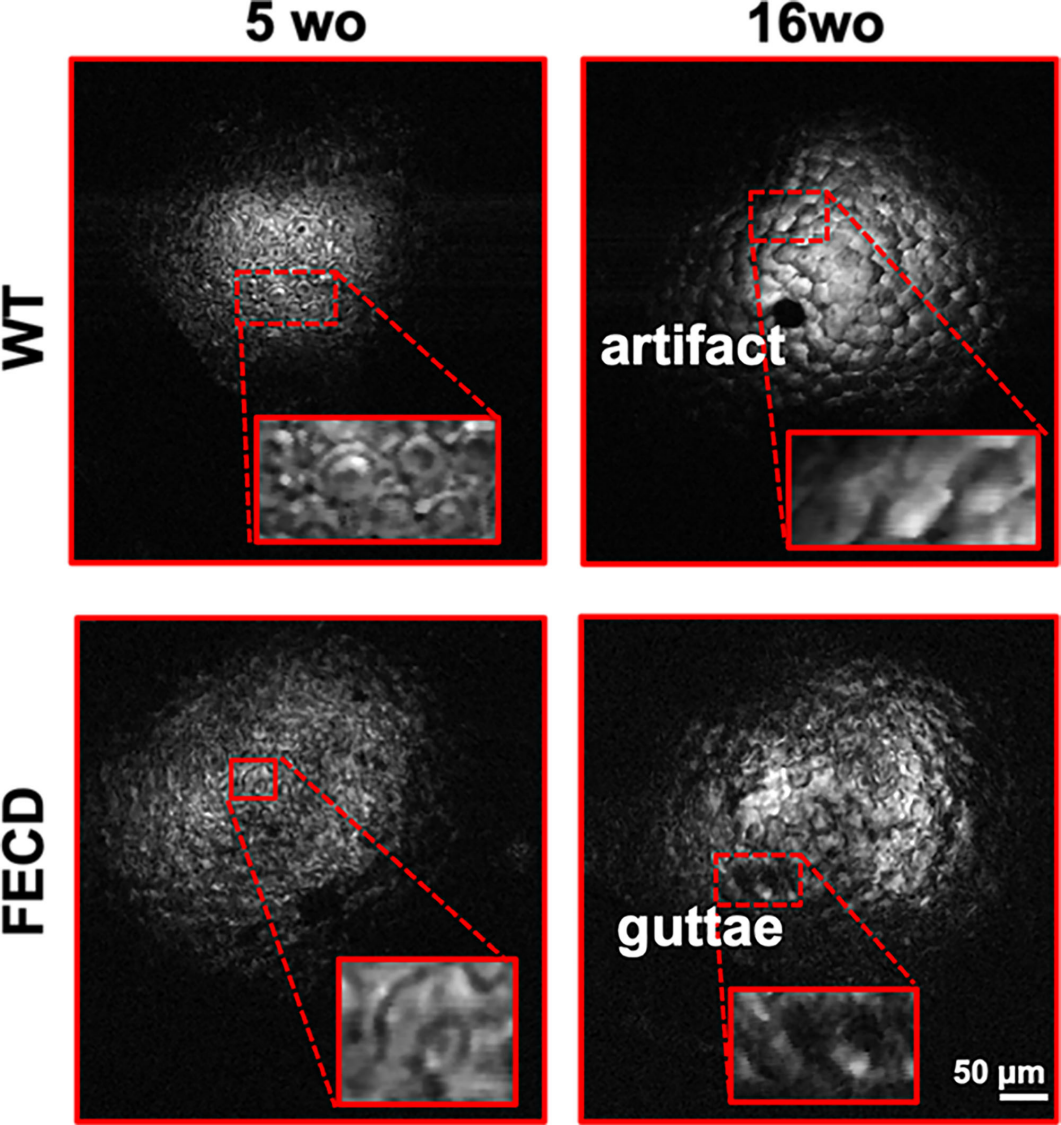
CECs were imaged using self-referenced OCM in WT and FECD mice at 5 weeks of age and 16 weeks of age. *Insets* indicate magnified views of the endothelial cells and guttae at 16 weeks of age FECD. The dark spot in the image at 16 weeks of age WT is caused by an artifact from the epithelium (possibly debris), resulting in a shadow extending throughout the depth of the cornea.

Figure 7 presents an example of en face images of endothelial cells obtained using self-referenced OCM in both WT and FECD mice at 5 weeks of age and 16 weeks of age. Notably, we observed the presence of guttae in double mutant FECD mice at 16 weeks of age, a key feature of FECD. This finding is consistent with the findings previously reported in Murugan et al.^14^ Note that the dark spot seen at 16 weeks of age in a WT mouse was an artifact (debris) from the corneal surface, which was visible throughout the depth of the cornea. The self-referenced approach provided enhanced structural detail and better contrast compared with the standard interference approach (not presented).

## Limitations of the Study

The measured axial resolution from self-referenced OCM was slightly higher than the theoretical value of 1.9 µm in air. This discrepancy can be attributed to the low quantum efficiency of the current line camera in the longer wavelength range.

One challenge of the proposed self-referenced technique was achieving optimal alignment of the steep mouse cornea with the system to enable real-time visualization of the tissue. The corneal surface must be positioned perpendicular to the objective in both the *x* and *y* axes (translation + rotation) to maximize the specular reflection from the corneal surface necessary to optimize the self-referenced interference signal. This required incremental adjustments of the mouse eye until the self-referenced image became visible. It was also necessary to manually adjust the Z-stage to optimize the focusing of the beam onto the DM and enable its segmentation. Not only were these manual alignment processes tedious, but they could potentially introduce interoperator variabilities in the image contrast and compromise the segmentation of the DM. Future work will integrate motorized stages and a feedback loop in the alignment process to automatically adjust the alignment of the mouse to the imaging system, thereby mitigating interoperator variabilities in image contrast, as well as potential segmentation errors caused by breathing-induced motion artifacts.

In addition, the corneal surface in mice was not uniformly smooth to generate a strong specular reflection for the self-referenced approach. Although Figure 7 presents qualitative images of endothelial cells and guttae in WT and FECD mice, the contrast of visible cells and the area of the region of interest were limited to objectively evaluate quantitative metrics. Future work will investigate strategies to further enhance the contrast of endothelial cells and the FOV of the system, which will enable the extraction of key quantitative metrics of endothelial cells, including cell density, hexagonality, and cell size. This quantitative analysis of cells will further enhance the capabilities of OCM for longitudinal studies. One strategy for enhancing image contrast will consist of using a mouse contact lens instead of saline solution. Indeed, although saline solution was helpful in mitigating corneal surface irregularity and dryness, its distribution was not uniform across the corneal surface to generate a consistent specular reflection, leading to image contrast degradation.

Although the self-referenced approach offered a slightly larger FOV than the standard reference approach, the region of interest was still limited to the center of the cornea, owing to the steep curvature of the mouse cornea. One strategy for enhancing the FOV will consist of adapting the hypercentric scanning approach described by Ruggeri et al.^31^ This scanning approach maintains the beam nearly perpendicular to the corneal surface across a wide lateral range, thereby enhancing the visibility of endothelial cells over a wide FOV, particularly at the periphery, where conventional telecentric scanning approaches struggle to collect backscattered light from steep corneal locations.

Another limitation of the study was the fact that two WT and two FECD mice had incomplete data at 5 weeks of age owing to image artifacts, and their data were replaced with data from mice of the same colony imaged at 5 weeks of age. Unfortunately, these replacement mice were sacrificed thereafter and could not be followed at 16 weeks owing to logistical constraints. This replacement has limited the longitudinal power of the experiment. As a result, true longitudinal tracking was achieved for two WT and three FECD mice. Nevertheless, although the inclusion of data at 5 weeks of age from these four additional mice may bias longitudinal inferences, it was interesting to note that the measured values in these replacement mice were within the measurement uncertainty range from other mice, supporting the reproducibility of this proof-of-concept experiment.

## Conclusions

To the best of our knowledge, this proof-of-concept study represents the first longitudinal in vivo evaluation of age-dependent changes in DM thickness in both WT and FECD mice using self-referenced OCM. Although the sample size and imaging timepoints were limited to infer conclusive findings, this pilot study offers tremendous opportunities for future longitudinal studies, especially given that OCM has been integrated with a custom-designed dual-channel confocal fluorescence microscopy sensitive to green and red fluorophores. Future studies will increase the sample size and include additional imaging time points to more precisely characterize age-dependent changes in DM thickness within individual animals. Further improvements in image contrast of endothelial cells will also enable to evaluate key quantitative metrics such as cell density, hexagonality, and cell size, which will further empower the capabilities of the system for longitudinal studies in the living mouse eye.

## References

[1] Singh JS, Haroldson TA, Patel SP. Characteristics of the low density corneal endothelial monolayer. *Exp Eye Res*. 2013; 115: 239–245. 23830909 10.1016/j.exer.2013.06.024

[2] Wilson SE, Bourne WM, O'Brien PC, Brubaker RF. Endothelial function and aqueous humor flow rate in patients with Fuchs' dystrophy. *Am J Ophthalmol*. 1988; 106: 270–278. 3262306 10.1016/0002-9394(88)90360-1

[3] Bonanno JA. Identity and regulation of ion transport mechanisms in the corneal endothelium. *Prog Retin Eye Res*. 2003; 22: 69–94. 12597924 10.1016/s1350-9462(02)00059-9

[4] Zhang W, Li H, Ogando DG, et al. Glutaminolysis is essential for energy production and ion transport in human corneal endothelium. *EBioMedicine*. 2017; 16: 292–301. 28117276 10.1016/j.ebiom.2017.01.004 PMC5474426

[5] Jeang L, Cha BJ, Birk DE, Espana EM. Endothelial-stromal communication in murine and human corneas. *Anat Rec (Hoboken)*. 2020; 303: 1717–1726. 32243086 10.1002/ar.24393 PMC8320741

[6] Jalimarada SS, Ogando DG, Bonanno JA. Loss of ion transporters and increased unfolded protein response in Fuchs’ dystrophy. *Mol Vis*. 2014; 20: 1668. 25548511 PMC4265779

[7] Murphy C, Alvarado J, Juster R, Maglio M. Prenatal and postnatal cellularity of the human corneal endothelium. A quantitative histologic study. *Invest Ophthalmol Vis Sci*. 1984; 25: 312–322. 6698749

[8] Singh RB, Parmar UPS, Kahale F, et al. Prevalence and economic burden of Fuchs endothelial corneal dystrophy in the Medicare population in the United States. *Cornea* 2024; 43: 1022–1027. 37906001 10.1097/ICO.0000000000003416

[9] Altamirano F, Ortiz-Morales G, O'Connor-Cordova MA, et al. Fuchs endothelial corneal dystrophy: an updated review. *Int Ophthalmol*. 2024; 44: 61. 38345780 10.1007/s10792-024-02994-1

[10] Aiello F, Gallo Afflitto G, Ceccarelli F, et al. Global prevalence of Fuchs endothelial corneal dystrophy (FECD) in adult population: A systematic review and meta-analysis. *J Ophthalmol*. 2022; 2022: 3091695. 35462618 10.1155/2022/3091695 PMC9023201

[11] Jun AS, Chakravarti S, Edelhauser HF, Kimos M. Aging changes of mouse corneal endothelium and Descemet's membrane. *Exp Eye Res*. 2006; 83: 890–896. 16777092 10.1016/j.exer.2006.03.025

[12] Bonanno JA, Shyam R, Choi M, Ogando DG. The h(+) transporter slc4a11: roles in metabolism, oxidative stress and mitochondrial uncoupling. *Cells*. 2022; 11: 197. 35053313 10.3390/cells11020197 PMC8773465

[13] Jalimarada SS, Ogando DG, Bonanno JA. Loss of ion transporters and increased unfolded protein response in Fuchs' dystrophy. *Mol Vis*. 2014; 20: 1668–1679. 25548511 PMC4265779

[14] Murugan S, de Campos VS, Ghag SA, et al. Characterization of a novel mouse model for Fuchs endothelial corneal dystrophy. *Invest Ophthalmol Vis Sci*. 2024; 65: 18. 10.1167/iovs.65.4.18 PMC11005065 38587441

[15] Vedana G, Villarreal G Jr., Jun AS. Fuchs endothelial corneal dystrophy: current perspectives. *Clin Ophthalmol*. 2016; 10: 321–330. 26937169 10.2147/OPTH.S83467 PMC4762439

[16] Matthaei M, Hu J, Meng H, et al. Endothelial cell whole genome expression analysis in a mouse model of early-onset Fuchs' endothelial corneal dystrophy. *Invest Ophthalmol Vis Sci*. 2013; 54: 1931–1940. 23449721 10.1167/iovs.12-10898 PMC3604908

[17] Schmedt T, Silva MM, Ziaei A, Jurkunas U. Molecular bases of corneal endothelial dystrophies. *Exp Eye Res*. 2012; 95: 24–34. 21855542 10.1016/j.exer.2011.08.002 PMC3273549

[18] Thériault M, Gendron SP, Brunette I, et al. Function-related protein expression in Fuchs endothelial corneal dystrophy cells and tissue models. *Am J Pathol.* 2018; 188: 1703–1712. 29698634 10.1016/j.ajpath.2018.03.014

[19] Gottsch JD, Bowers AL, Margulies EH, et al. Serial analysis of gene expression in the corneal endothelium of Fuchs' dystrophy. *Invest Ophthalmol Vis Sci*. 2003; 44: 594–599. 12556388 10.1167/iovs.02-0300

[20] Ogando DG, Shyam R, Kim ET, et al. Inducible slc4a11 knockout triggers corneal edema through perturbation of corneal endothelial pump. *Invest Ophthalmol Vis Sci*. 2021; 62: 28. 10.1167/iovs.62.7.28 PMC8826551 34190974

[21] Cai Z, Zhang Y, Fang RS, et al. Multiscale imaging of corneal endothelium damage and rho-kinase inhibitor application in mouse models of acute ocular hypertension. *Biomed Opt Express*. 2024; 15: 1102–1114. 38404323 10.1364/BOE.510432 PMC10890882

[22] Inomata T, Mashaghi A, Hong J, et al. Scaling and maintenance of corneal thickness during aging. *PloS One*. 2017; 12: e0185694. 28985226 10.1371/journal.pone.0185694 PMC5630165

[23] Canavesi C, Cogliati A, Mietus A, et al. In vivo imaging of corneal nerves and cellular structures in mice with Gabor-domain optical coherence microscopy. *Biomed Opt Express*. 2020; 11: 711–724. 32133220 10.1364/BOE.379809 PMC7041447

[24] Ang M, Konstantopoulos A, Goh G, et al. Evaluation of a micro-optical coherence tomography for the corneal endothelium in an animal model. *Sci Rep*. 2016; 6: 29769. 27416929 10.1038/srep29769 PMC4945948

[25] Maddipatla R, Tankam P. Development of high-speed, integrated high-resolution optical coherence microscopy and dual-channel fluorescence microscopy for the simultaneous co-registration of reflectance and fluorescence signals. *Optics and Lasers in Engineering*. 2022; 149: 106823.

[26] Messner A, Fischak C, Pfister M, et al. Characterization of dry eye disease in a mouse model by optical coherence tomography and fluorescein staining. *Biomed Opt Express*. 2019; 10: 4884–4895. 31565532 10.1364/BOE.10.004884 PMC6757454

[27] Chen S, Liu X, Wang N, et al. Visualizing micro-anatomical structures of the posterior cornea with micro-optical coherence tomography. *Sci Rep*. 2017; 7: 10752. 28883661 10.1038/s41598-017-11380-0 PMC5589810

[28] Bell BA, Kaul C, Hollyfield JG. A protective eye shield for prevention of media opacities during small animal ocular imaging. *Exp Eye Res*. 2014; 127: 280–287. 25245081 10.1016/j.exer.2014.01.001 PMC4173074

[29] Krstajić N, Brown CT, Dholakia K, Giardini ME. Tissue surface as the reference arm in Fourier domain optical coherence tomography. *J Biomed Opt*. 2012; 17: 071305. 22894466 10.1117/1.JBO.17.7.071305

[30] Monfort T, Azzollini S, Ben Yacoub T, et al. Interface self-referenced dynamic full-field optical coherence tomography. *Biomed Opt Express*. 2023; 14: 3491–3505. 37497503 10.1364/BOE.488663 PMC10368024

[31] Ruggeri M, Giuffrida FP, Vy Truong NL, et al. Wide-field self-referenced optical coherence tomography imaging of the corneal microlayers. *Opt Lett*. 2025; 50: 1204–1207. 39951764 10.1364/OL.542289 PMC12691542

[32] Pattan HF, Liu X, Tankam P. Non-invasive in vivo imaging of human corneal microstructures with optical coherence microscopy. *Biomed Opt Express*. 2023; 14: 4888–4900. 37791273 10.1364/BOE.495242 PMC10545177

[33] Pattan HF, Liu X, Tankam P. In vivo assessment of human corneal epithelial cells in orthokeratology lens wearers: a pilot study. *Optom Vis Sci*. 2024; 101: 263–271. 38683973 10.1097/OPX.0000000000002130

[34] Receno CN, Eassa BE, Cunningham CM, DeRuisseau LR. Young and middle-aged mouse breathing behavior during the light and dark cycles. *Physiol Rep*. 2019; 7: e14060. 31004390 10.14814/phy2.14060 PMC6474843

[35] de Kinkelder R, Kalkman J, Faber DJ, et al. Heartbeat-induced axial motion artifacts in optical coherence tomography measurements of the retina. *Invest Ophthalmol Vis Sci*. 2011; 52: 3908–3913. 21467182 10.1167/iovs.10-6738

[36] Farrelly O, Suzuki-Horiuchi Y, Brewster M, et al. Two-photon live imaging of single corneal stem cells reveals compartmentalized organization of the limbal niche. *Cell Stem Cell*. 2021; 28: 1233–1247.e4. 33984283 10.1016/j.stem.2021.02.022 PMC8559309

[37] Tankam P, Pattan HF, Dhaliwal DK. Multifocal beam in self-referenced optical coherence microscopy for non-contact in vivo imaging of human corneal endothelial cells. *Opt Lett*. 2025; 50: 4518–4521.

[38] Patel SV, McLaren JW. In vivo confocal microscopy of Fuchs endothelial dystrophy before and after endothelial keratoplasty. *JAMA Ophthalmol*. 2013; 131: 611–618. 23471204 10.1001/jamaophthalmol.2013.799

[39] Coco G, Levis HJ, Borgia A, et al. Posterior stromal ripples increase risk of Descemet's membrane endothelial keratoplasty graft detachment worsening over time. *Acta Ophthalmol*. 2023; 101: e205–e214. 36120722 10.1111/aos.15250

[40] Ventura M, Airaldi M, Ancona C, et al. Preoperative posterior stromal ripples as predictive biomarkers of visual recovery after Descemet membrane endothelial keratoplasty. *Cornea*. 2025; 44: 976–982. 10.1097/ICO.0000000000003698 PMC12208380 39450998

[41] Johnson DH, Bourne WM, Campbell RJ. The ultrastructure of Descemet's membrane: I. Changes with age in normal corneas. *Arch Ophthalmol*. 1982; 100: 1942–1947. 7150061 10.1001/archopht.1982.01030040922011

[42] Zhang J, Patel DV. The pathophysiology of Fuchs' endothelial dystrophy–a review of molecular and cellular insights. *Exp Eye Res*. 2015; 130: 97–105. 25446318 10.1016/j.exer.2014.10.023

